# Emergency Department Visit Data for Rapid Detection and Monitoring of Norovirus Activity, United States

**DOI:** 10.3201/eid1908.130483

**Published:** 2013-08

**Authors:** Brian Rha, Sherry Burrer, Soyoun Park, Tarak Trivedi, Umesh D. Parashar, Benjamin A. Lopman

**Affiliations:** Centers for Disease Control and Prevention, Atlanta, Georgia, USA (B. Rha, S. Burrer, S. Park, T. Trivedi, U.D. Parashar, B.A. Lopman);; McKing Consulting Corporation, Atlanta (S. Park)

**Keywords:** norovirus, biosurveillance, outbreaks, gastroenteritis, rotavirus, viruses, surveillance, emergency department, United States, chief complaint, tracking, detection, enteric infections

## Abstract

Noroviruses are the leading cause of gastroenteritis in the United States, but timely measures of disease are lacking. BioSense, a national-level electronic surveillance system, assigns data on chief complaints (patient symptoms) collected during emergency department (ED) visits to 78 subsyndromes in near real-time. In a series of linear regression models, BioSense visits mapped by chief complaints of diarrhea and nausea/vomiting subsyndromes as a monthly proportion of all visits correlated strongly with reported norovirus outbreaks from 6 states during 2007–2010. Higher correlations were seen for diarrhea (*R* = 0.828–0.926) than for nausea/vomiting (*R* = 0.729–0.866) across multiple age groups. Diarrhea ED visit proportions exhibited winter seasonality attributable to norovirus; rotavirus contributed substantially for children <5 years of age. Diarrhea ED visit data estimated the onset, peak, and end of norovirus season within 4 weeks of observed dates and could be reliable, timely indicators of norovirus activity.

Noroviruses are the most common cause of epidemic and sporadic gastroenteritis worldwide (*1*–*4*). In the United States, norovirus gastroenteritis causes an estimated 21 million cases of illness and ≈800 deaths annually (*5*,*6*), resulting in an estimated 1.7 million physician’s office visits, 400,000 emergency department (ED) visits, and 71,000 hospitalizations each year. The estimated annual cost for norovirus-related health care in the United States is $777 million (*7*,*8*).

Timely monitoring of norovirus activity has remained elusive in part because of the scarcity of diagnostic testing for patients with suspected disease. Approximately 90% of persons with acute viral gastroenteritis do not seek medical attention; of those who do, only 6% submit stool specimens for diagnostic testing, in part because testing is often not deemed necessary for self-limited illness (*2*). Furthermore, no rapid and sensitive clinical assay is widely available in the United States, and definitive diagnosis requires PCR, which is used primarily in public health laboratories; therefore, few nonoutbreak cases are laboratory confirmed (*9*). As a result, existing US norovirus surveillance depends on outbreak investigations that can be subject to substantial delays in reporting. This process of notifying local/state health departments of disease, subsequently investigating the outbreak, testing specimens, and voluntarily reporting to the national surveillance system can vary widely in duration (days to months), which makes timely and uniform monitoring on a national level challenging (*9*,*10*).

The timing and magnitude of norovirus seasonal activity varies from year to year (*11*,*12*). Timely monitoring could rapidly identify the season onset and elevated levels of activity, which could potentially improve prevention and control efforts by public health and infection control personnel in health care settings, help with planning for increased health care utilization in facilities, and alert the public with timely prevention messages. Syndromic surveillance data based on ED visits related to gastroenteritis might be robust and timely surrogate measures of norovirus activity, given its characteristic wintertime seasonality.

BioSense is a timely, national-level electronic health surveillance system that receives and processes healthcare encounter data to conduct syndromic surveillance and is maintained by the Centers for Disease Control and Prevention. A subset of the data that BioSense receives is ED visit data: codes from the International Classification of Diseases, 9th Revision, Clinical Modification (www.cdc.gov/nchs/icd/icd9cm.htm), and chief complaint (i.e., patient-reported symptoms) information entered in text format. These data are then mapped in near real-time to 15 syndromes and 78 subsyndromes, including those related to gastroenteritis symptoms (*11*,*12*). For this study, we assessed whether BioSense chief complaint–based ED visit data could be a reliable indicator of norovirus activity in the United States by determining the degree of correlation of these data with reported norovirus outbreaks.

## Methods

### Data Sources

#### BioSense Chief Complaint Data

ED chief complaint–based data used in analyses were collected and processed daily in the BioSense 1.0 platform from >600 participating nonfederal hospitals in 26 states during January 2005–June 2011. In 2008, the median time from patient visit to chief complaint data receipt was 4 hours (*13*). Daily counts of ED visits that mapped to either of 2 BioSense subsyndromes, “Diarrhea” or “Nausea and Vomiting” (hereafter as diarrhea and nausea/vomiting), on the basis of chief complaint text were aggregated into weekly counts by state and age group. For example, any visit with a chief complaint text field featuring keywords such as “nausea” or “vomiting,” as well as associated abbreviations and misspellings, was mapped to the nausea/vomiting subsyndrome. Diarrhea and nausea/vomiting visits were not necessarily mutually exclusive, because a given chief complaint text substring can be assigned to >1 subsyndrome by the BioSense system (*13*). Weekly counts of all-cause ED visits by state and age group were also compiled so that the proportion of ED visits mapped to each subsyndrome could be calculated.

#### Norovirus Outbreak Surveillance Data

Monthly norovirus outbreak counts were based on reports of suspected and confirmed norovirus outbreaks elicited from 30 states during January 2007–April 2010 (*12*); these monthly data were used as the main comparison for the BioSense data. For a separate analysis, weekly norovirus outbreak surveillance data were obtained from the National Outbreak Reporting System (NORS), a national-level, Internet-based reporting platform established in 2009 (*10*). These weekly data were based on suspected and confirmed norovirus outbreaks as reported by 47 state and territory health departments during January 2009–December 2011.

#### Rotavirus Laboratory Surveillance Data

To control for the possible contribution of rotavirus, another diarrheal pathogen with distinct winter seasonality, we obtained data reported to the National Respiratory and Enteric Virus Surveillance System by participating laboratories in 24 states during January 2006–June 2011. These laboratories report data aggregated on a weekly basis that includes the total number of rotavirus tests performed and the number of tests that were positive for rotavirus.

### Data Analyses

#### Statistical Methods

Linear regression models were fitted to assess the degree of correlation between syndromic surveillance ED visit data from the BioSense system and norovirus outbreak activity following an approach that has been described (*14*). In a series of linear regression models, BioSense ED visits mapped by chief complaint to diarrhea and nausea/vomiting (as a monthly proportion of all ED visits) were regressed on monthly reported norovirus outbreaks from January 2007–April 2010. We restricted analysis to the 6 states (Georgia, Missouri, Ohio, Pennsylvania, Tennessee, and Wyoming) that had uninterrupted data during that time for 1) BioSense, 2) monthly norovirus outbreaks, and 3) rotavirus antigen tests (>120 tests/year). For these analyses, weekly BioSense and rotavirus test data were aggregated by month. Linear regression models were fitted separately for 5 age groups (0–4, 5–17, 18–64, >65 years, and all ages). To avoid attributing secular trends in syndromic data to norovirus, a sequential variable for month of study was included in the models. In addition, a term for laboratory-reported test data for rotavirus, a major cause of gastroenteritis in those <5 years of age, was also included in the model initially. For the sake of parsimony, the rotavirus term was subsequently removed from models in which its coefficient was not significant or positive in preliminary analyses. 

The models can be expressed with the following formulas:

pCC*_x,y_* = α + (β_1_ × Noro*_y_*) + (β_2_ × Rota*_y_*) + (β_3_ × Time*_y_*) for models in which the rotavirus term was significant and positive, and

pCC*_x,y_* = α + (β_1_ × Noro*_y_*) + (β_2_ × Time*_y_*) for all other models, where pCC is the proportion of ED visits mapped by chief complaint to the subsyndrome of interest, Noro is the count of reported norovirus outbreaks, Rota is the proportion of positive rotavirus antigen tests, in age group *x* and year-month *y*, the intercept α represents the background proportion of ED visits mapped by chief complaint to the subsyndrome of interest, and Time is the sequential variable for month of study included to account for secular trends. These models assumed that the relationships between BioSense data and 1) norovirus outbreak and 2) rotavirus test data were constant over time. The Pearson correlation coefficient (*R*) and the coefficient of determination (*R*^2^) were calculated for each model across the 5 age groups to determine the strength and direction of the relationship between ED visits mapped by chief complaint to diarrhea or nausea/vomiting and norovirus outbreak data.

The proportions of diarrhea-related visits predicted to be attributable to norovirus, rotavirus, background, or secular trends for each month were estimated by multiplying the monthly values for each predictor with its corresponding coefficient in the regression model. The proportions of diarrhea visits attributable to background and secular trends were combined to establish a nonseasonal baseline proportion of diarrhea visits with other etiologies. To determine whether the relationship between BioSense ED visit data and monthly norovirus outbreaks was robust at the state level, linear regression models for the all-ages group were fitted separately for each of the 6 states.

Microsoft Excel (Microsoft, Redmond, WA, USA) and SAS version 9.3 (SAS Institute, Inc., Cary, NC, USA) software were used to perform all analyses. Assumptions of linearity, common variance, and normality were checked by visual inspection of residual plots for all models.

#### Estimating Norovirus Seasonal Time Markers

To determine if BioSense ED visit data could be used to predict norovirus season markers (i.e., onset, peak, and end), we compared weekly diarrhea visits mapped by chief complaint as a proportion of all visits (for all ages) with weekly norovirus outbreak data from NORS for the 2009–2011 seasons on a national level. All states/territories that contributed data to BioSense (n = 26) and/or NORS (n = 46) during those seasons were included in this analysis. Outbreak data from NORS were used because they are weekly, which allowed for detection of seasonal time markers at the week level for these 2 seasons. First, NORS data were used to identify the onset, peak, and end weeks of the 2009–2010 norovirus season (weeks ending July 11, 2009–July 3, 2010). Season onset was defined as the week by which at least 10% of the year’s cumulative total number of outbreaks had occurred, and season end was defined as the week by which at least 90% of the year’s cumulative total number of outbreaks had occurred. The peak was defined as the week with the highest number of outbreaks. Using these definitions, we then used the observed onset, peak, and end week dates to formulate rules by which corresponding diarrhea visit data could be used to estimate the season’s onset, peak, and end. These rules were then applied to 2010–2011 data (weeks ending July 10, 2010–July 2, 2011) to test their performance in estimating the time markers for that season.

## Results

### Correlation

**Figure 1:**
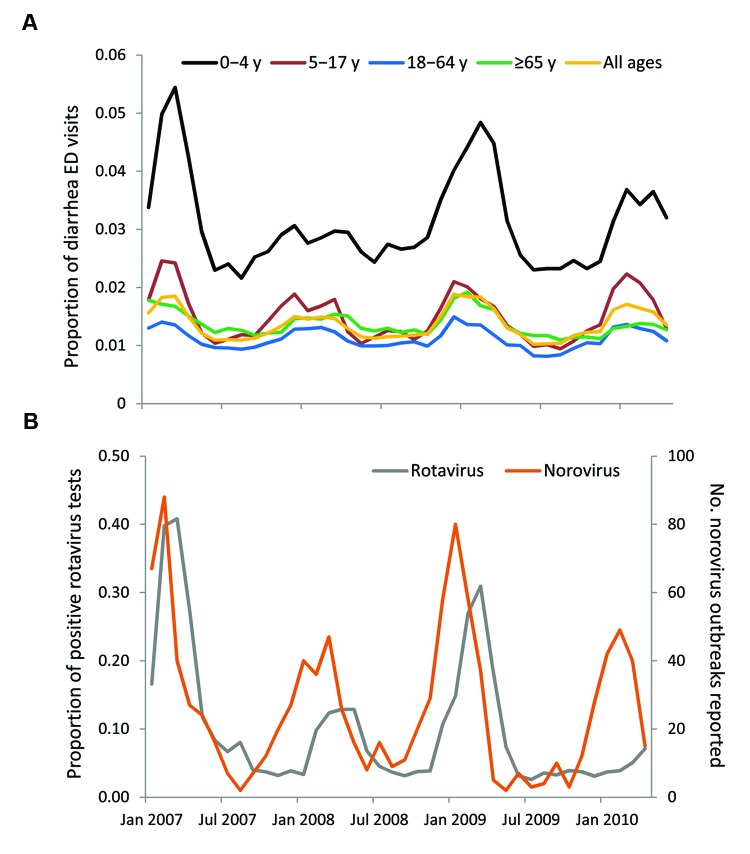
Proportion of BioSense emergency department (ED) visits for diarrhea subsyndrome (A) and norovirus and rotavirus surveillance data (B), United States, January 2007–April 2010. The proportion of ED visits mapped by chief complaint to diarrhea subsyndrome in the 6 states analyzed (Georgia, Missouri, Ohio, Pennsylvania, Tennessee, and Wyoming) and reported norovirus outbreak data displayed seasonal peaks in the winter months. This seasonal pattern was observed for all 5 age groups (0–4, 5–17, 18–64, >65 years, and all ages); a higher proportion of ED visits for diarrhea was seen among children <5 years of age. Rotavirus activity, as measured by the proportion of positive antigen tests, also showed winter seasonality, with peaks that lagged behind those of norovirus.

During January 2007–April 2010, the 6 states analyzed reported 1,048 norovirus outbreaks and 32,455 rotavirus antigen tests (4,197 [13%] with positive results). During the same period, BioSense received data from the 6 states for 20,205,284 total ED visits, of which 277,433 (1.4%) were diarrhea and 1,165,414 (5.8%) were nausea/vomiting visits. More than half (56%) of all ED visits were by patients in the 18–64-year age group. For each month, the proportion of ED diarrhea visits was higher for children <5 years of age (0.022–0.054) than for persons in any other age group (0.008–0.025). Over the 40-month period, a seasonal pattern was observed in the proportion of diarrhea visits for each age group that mirrored the seasonal variation observed in the reported norovirus outbreaks, with peaks in the winter months (Figure 1). The proportion of positive rotavirus test results peaked 1–2 months after the peaks of norovirus outbreaks.

The monthly proportion of visits for diarrhea or nausea/vomiting had strong linear relationships with norovirus outbreaks for each age group (p<0.001), with stronger correlations for diarrhea visits (*R* = 0.828–0.926) than for nausea/vomiting visits (*R* = 0.729–0.866) (Table 1). The proportion of positive rotavirus test results was significant and positive in the diarrhea and nausea/vomiting models for the 0–4-year age group, as were diarrhea models for the >65-year and all-ages groups (p<0.05). The time variable was kept in the models to give conservative estimates of the contribution of norovirus outbreaks, even though it was not significant for all models. Although a few models showed possible evidence of skew at extreme values, we kept our linear models for the sake of parsimony and used diarrhea models for all subsequent analyses because diarrhea had higher correlation across all age groups. The best-fit models were for diarrhea in the 0–4-year, >65-year, and all-ages groups (*R*^2^ = 0.829–0.857). Overall, the models indicate that the seasonal variation by month in the proportion of diarrhea visits as reported by BioSense can be attributed to reported norovirus outbreak activity, with rotavirus also playing a role in the 0–4-year, >65-year, and all-ages groups.

**Table 1:** Linear regression model estimates of the association between norovirus outbreaks and BioSense emergency department visit data, by age group, United States, January 2007–April 2010*

Subsyndrome and age group, y	Norovirus,† β_1_, × 10^−4^ (95% CI)	p value‡	*R*
Diarrhea			
0–4§	1.72 (1.15–2.29)	<0.0001	0.926
5–17¶	1.59 (1.23–1.95)	<0.0001	0.828
18–64¶	0.70 (0.56–0.83)	<0.0001	0.864
>65§¶	0.71 (0.55–0.86)	<0.0001	0.917
All ages§	0.94 (0.74–1.14)	<0.0001	0.910
Nausea/vomiting			
0–4§	7.01 (4.93–9.09)	<0.0001	0.866
5–17	5.73 (4.24–7.23)	<0.0001	0.796
18–64¶	1.60 (1.14–2.06)	<0.0001	0.758
>65	0.61 (0.31–0.91)	0.0002	0.729
All ages	2.78 (2.15–3.40)	<0.0001	0.832

*Emergency department chief complaint–based subsyndrome visits as a monthly proportion of all visits in 6 states (GA, MO, OH, PA, TN, WY) regressed on norovirus and rotavirus surveillance data and time variable. Intercept for each model p<0.0001. †Suspected and confirmed norovirus outbreaks. ‡By *t* test. §Included term for proportion of rotavirus tests positive in the model, as it is significant and positive (p<0.05). β_2_ for 0–4-y age group diarrhea model = 0.0539 (95% CI 0.0403–0.0674); β_2_ for 0–4-y age group nausea/vomiting model = 0.0745 (95% CI 0.0252–0.1237); β_2_ for ≥65-y age group diarrhea model = 0.0048 (95% CI 0.0011–0.0084); β_2_ for all-ages age group diarrhea model = 0.0065 (95% CI 0.0018–0.0112). ¶Time variable not significant in model (p>0.05).

### Predicted Contribution of Norovirus/Rotavirus to BioSense Activity

**Figure 2:**
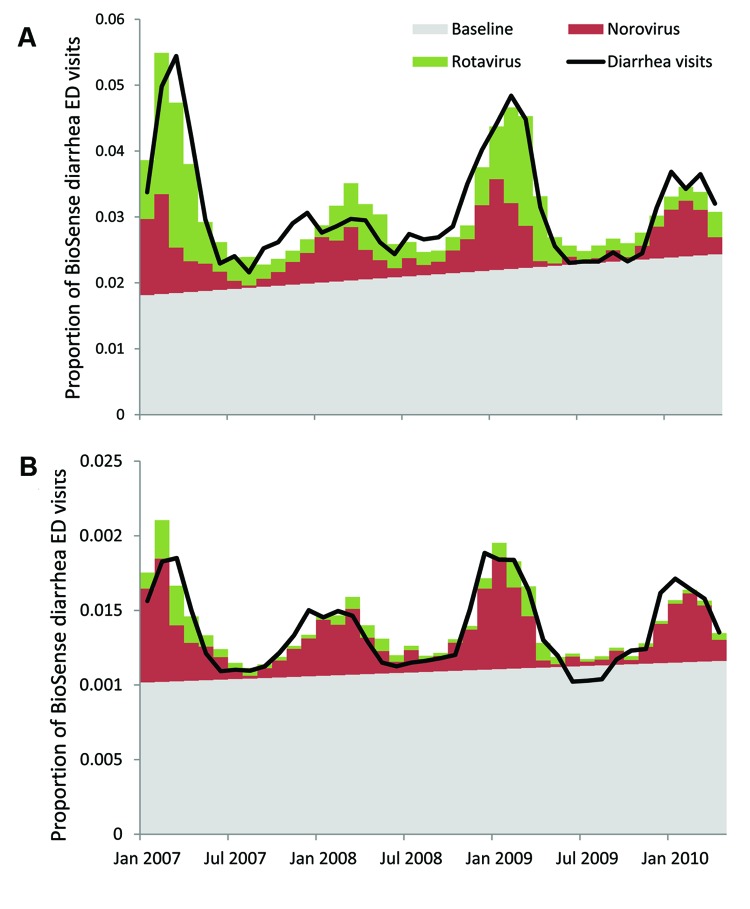
Model attribution of the proportion of BioSense emergency department (ED) visits mapped by chief complaint to diarrhea subsyndrome compared with estimates of norovirus and rotavirus infections, United States, January 2007–April 2010. A) Patients 0–4 years of age; B) patients of all ages. Predicted norovirus largely accounted for the observed seasonal variations in the proportion of diarrhea visits in the all-ages group (17.5% of predicted total, January 2007–April 2010), with rotavirus making a smaller contribution (4.7%); norovirus (13.6%, August 2007–April 2010) and predicted rotavirus (13.6%) equally accounted for the seasonality in the 0–4-year age group. All other etiologies captured by the background and secular increase (baseline) did not contribute to the observed winter seasonality.

The models predicted a linear baseline proportion of diarrhea visits that occur year-round, above which seasonal variation that is observed can largely be attributed to predicted values of norovirus (for all ages) or norovirus and rotavirus (for the 0–4-year age group) (Figure 2). For patients of all ages, norovirus was estimated to account for 17.5% of the predicted proportion of BioSense diarrhea visits over the 40-month span (rotavirus accounted for 4.7%), or 23.9% when restricting analysis to months that typically encompass the norovirus season (November–April). For children <5 years of age, we excluded data before August 2007 to capture the contribution of norovirus and rotavirus after the introduction of rotavirus vaccination. Norovirus accounted for 13.6% of the predicted proportion of diarrhea visits among children in this age group and rotavirus accounted for 13.7%.

### State-level Robustness

**Figure 3:**
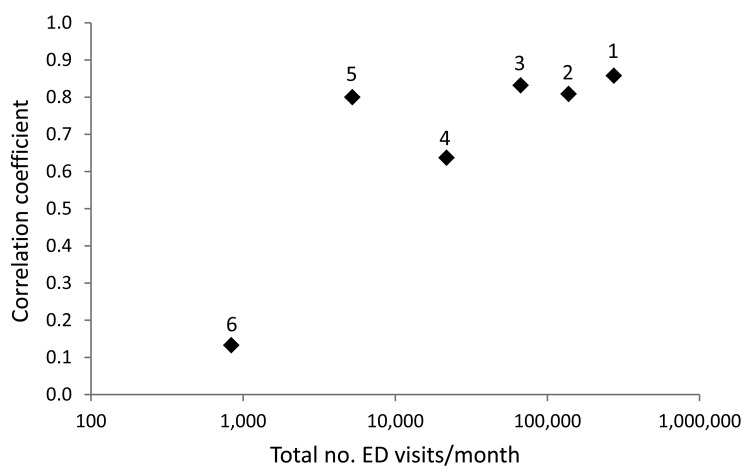
Correlation between the proportion of BioSense emergency department (ED) visits mapped by chief complaint to diarrhea subsyndrome and norovirus outbreaks as a function of total BioSense ED visits per month using state-specific data for the 6 states analyzed, United States, January 2007–April 2010. Correlation coefficients for each state are plotted by corresponding total ED visits/month on a logarithmic scale. Models tended to perform better in states with greater total ED visits. Higher correlation (*R*>0.60) was observed for states with >5,000 BioSense ED visits/month. State number labels on data points correspond to those in Table 2.

When considering the relationship between BioSense diarrhea visit data for all ages and reported norovirus outbreaks at the state level, correlation was generally higher in states with a greater number of recorded ED visits/month. *R*>0.60 was observed for all states with >5,000 BioSense ED visits/month (Figure 3; Table 2).

**Table 2:** Parameters of linear regression models of the association between norovirus outbreaks and BioSense emergency department diarrhea subsyndrome visit data, by state, United States, January 2007–April 2010*

State no.	Total no. emergency department visits/mo	Norovirus,† β_1_, × 10^−4^ (95% CI)	p value‡	*R*
1	273,218	2.86 (1.72 to 4.00)	<0.0001	0.858
2§	137,584	6.91 (4.60 to 9.22)	<0.0001	0.809
3¶	66,597	3.79 (2.74 to 4.83)	<0.0001	0.832
4¶	21,684	7.06 (0.73 to 13.40)	0.0298	0.637
5¶	5,214	2.18 (1.57 to 2. 78)	<0.0001	0.800
6§¶	836	0.53 (−8.77 to 9.84)	0.9082	0.133

*Emergency department chief complaint–based visits for diarrhea subsyndrome as a monthly proportion of all visits regressed on norovirus surveillance data, rotavirus antigen test data, and time variable. Intercept for each model p<0.0001. †Suspected and confirmed norovirus outbreaks. ‡By *t* test. ¶Time variable not significant in model (p>0.05). §Proportion of rotavirus tests positive variable not significant in model (p>0.05).

### Estimating Season Markers

**Figure 4:**
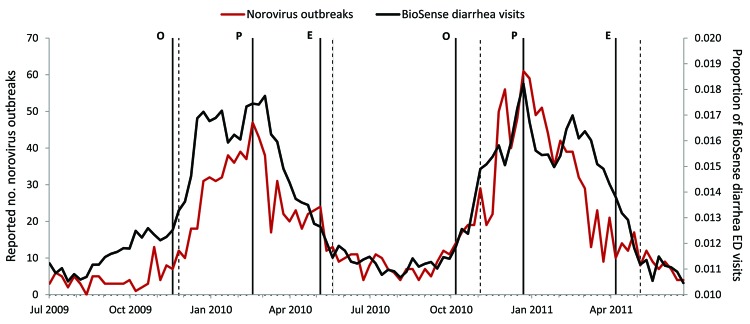
Estimation of norovirus season time markers using BioSense data on emergency department (ED) visits mapped by chief complaint to diarrhea subsyndrome, United States, 2009–2011. Observed season time markers (solid vertical lines) as defined by norovirus outbreak data are labeled as follows: season onset (O), season peak (P), and season end (E). Applying these rules yielded estimates for each season marker (dotted vertical lines) within 2 weeks of observed dates for the 2009–2010 season and within 4 weeks of observed dates for the 2010–2011 season.

By applying our definitions of norovirus seasonal time markers to the 2009–2010 season by using weekly NORS outbreak data, we observed the following season markers (by week/year): onset, 47/2009; peak, 8/2010; and end, 19/2010. On the basis of visual inspection of the all-ages weekly national BioSense data for diarrhea visits for the 2009–2010 season, we determined the following rules to define the onset, peak, and end: 1) norovirus season begins when the proportion of diarrhea ED visits is >0.0125 (1.25%) for 2 consecutive weeks, 2) season peak occurs when the proportion of diarrhea ED visits is >0.0170 (1.70%) for 2 consecutive weeks, and 3) season ends when the proportion of diarrhea ED visits is <0.0125 (1.25%) for 2 consecutive weeks. Applying these rules to the 2009–2010 season yielded estimates for each season marker within 2 weeks of the observed dates (Figure 4). The rules were then tested on NORS data from the 2010–2011 season, yielding estimates within 4 weeks of the following observed marker dates: onset, 41/2010; peak, 52/2010; and end, 15/2011. Notably, the proposed rules correctly predicted the earlier observed onset, peak, and end dates for the 2010–2011 season when compared with the previous season.

## Discussion

We found that BioSense syndromic data based on patient-reported symptoms (chief complaints) from ED visits correlated strongly with norovirus outbreak activity. These findings demonstrate that temporal signals in community syndromic surveillance time series can accurately reflect trends in norovirus activity across several regions and age groups. Specifically, the monthly proportion of BioSense ED diarrhea visits exhibited winter seasonality that mirrored that seen in reported norovirus outbreaks over 3 seasons, with the best-fit models accounting for >82% of the variation observed in the chief complaint–based syndromic data. Nausea/vomiting models did not fit as well as diarrhea models, which may be because the definition for the nausea/vomiting subsyndrome is less specific, as suggested by the fact that nausea/vomiting visits greatly outnumbered diarrhea visits. Our diarrhea models predict that much of the observed seasonal variation can be attributed to norovirus, with rotavirus also contributing substantially to seasonality in children <5 years of age. Nationally, this system has the potential to identify key attributes of the norovirus season (i.e., onset, peak, and end) in near real-time. The robustness of this relationship depends on the number of ED visits captured by the syndromic surveillance system; states with >5,000 recorded ED visits per month may be able to reliably use BioSense to accurately monitor community norovirus activity at the state level. Overall, our results suggest that BioSense chief complaint–based ED diarrhea visits can be a useful and timely indicator of norovirus disease in the United States.

The proportion of ED visits mapping to diarrhea exhibited a seasonality that can largely be explained by norovirus, which accounted for 17.5% of the overall ED activity for diarrhea. Although our estimates are based on proportions of diarrhea visits rather than exact counts, useful comparisons can still be made to norovirus prevalence reported for previous studies in the ED setting, including 18% for gastroenteritis patients of all ages (*8*) and 26% for adults (*15*). For children <5 years of age, our models suggest that norovirus (13.6%) and rotavirus (13.7%) caused similar proportions of diarrhea ED visits contributing to seasonal variation after rotavirus vaccination was introduced in the 2007–2008 season (*16*). This level of activity is lower than the norovirus prevalence (23%) reported in a recent US study through laboratory testing of children <5 years of age with gastroenteritis over 2 seasons from 2008–2010 (*17*) but more closely approximates the pooled proportion (12%, 95% CI 10%–15%) of children <5 years of age with severe diarrhea reported by a systematic review of 19 studies from low- to high-income countries (*18*). This winter seasonality is a well-described attribute of norovirus outbreaks (*19*,*20*) that has been used to estimate rates of norovirus-associated ED and ambulatory care visits, hospitalizations, and deaths (*6*–*8*,*21*–*23*). In addition, new variants of norovirus emerge every 3–5 years and are sometimes associated with surges in incidence (*11*), a pattern that was detected by a local ED-based syndromic surveillance system in Boston, Massachusetts, USA, during the winter of 2006–2007 (*24*). Our study expands on these observations by correlating ED-based syndromic signals with disease outcomes in a simple model.

The need for timely estimates of norovirus activity has led to previous efforts to develop surrogate measures of disease from other types of syndromic data. In the United Kingdom, an early warning system for norovirus activity based on the subjects of calls to a national telemedicine hotline gave up to 4 weeks advance warning of season onset (*25*). More recently, Internet search trends for terms related to gastroenteritis symptoms have been examined as timely surrogates for norovirus activity (*26*,*27*); in the United States, high correlation with outbreaks was demonstrated at national and regional levels (*26*). However, a limitation of Internet search data is their potential to be affected by media and social interest rather than disease incidence. Here we have shown ED visit data containing BioSense diarrhea subsyndrome chief complaints also have good potential for estimating norovirus season onset, as well as its peak and end, and have the advantage of being more closely linked to clinical outcome than are Internet search trends.

Certain limitations should be considered when interpreting our findings. First, chief complaint data, like all syndromic data, lack specificity in etiology. Our model acknowledges that other infectious and noninfectious illnesses can result in ED visits mapped to diarrhea by including a background term and only attributes the seasonal fraction to norovirus (and rotavirus in certain age groups). Although other pathogens that exhibit seasonality, such as astrovirus and sapovirus, were not accounted for in our model, these pathogens are detected at substantially lower rates than norovirus (*2*,*3*,*15*). 

A second limitation is that each facility’s catchment area and the duration of its participation during the study period were not known, so population-based rates could not be calculated. We accounted for facilities coming in and out of the system by using as our primary outcome the proportion of diarrhea visits. However, this measure may be subject to bias if the denominator (total all-cause ED visits) fluctuates for reasons independent of norovirus activity. For example, an increase in total ED visits because of respiratory illnesses would lower the proportion of diarrhea visits, even if the number of diarrhea visits remained unchanged. However, none of these considerations appear to diminish the value of using BioSense data as a timely indicator of norovirus activity. 

Finally, our analyses could not account for any local variations in model performance resulting from any other factor beyond the volume of visits. The strength of correlation did vary from state to state, but the overall high degree of correlation suggests that this is not a fundamental limitation. Indeed, expanding the scope of our analyses with national-level data to develop rules for predicting season onset, peak, and end yielded promising results that can be used as a starting point for further refinement and prospective validation in the future.

The impact of norovirus in the United States is becoming increasingly clear, but traditional surveillance has not been sufficiently timely in identifying aberrant activity. Although syndromic surveillance lacks specificity, the strength of correlation with reported norovirus outbreaks we observed highlights the value of these data for rapid detection of norovirus activity. On a practical level, early detection that the norovirus season has started can serve as warning to infection control practitioners and the general public and might also help to detect the emergence of novel norovirus strains with pandemic potential. In this way, the proposed surveillance method applied within BioSense and described here can serve as a useful adjunct to existing surveillance systems. The emergence of the GII.4 Sydney norovirus strain during the 2012–2013 season (*28*), in particular, serves as a reminder of the need for timely surveillance tools to assess the timing and magnitude of norovirus activity.
